# Late‐Onset Mesalazine‐Induced Eosinophilic Pneumonitis Presenting as Non‐Resolving Pneumonia: A Diagnostic Pitfall in Ulcerative Colitis

**DOI:** 10.1002/ccr3.72029

**Published:** 2026-02-16

**Authors:** Siraj Nasim, Tamer Mohamed Zaalouk, Abu Bocus, Maged Kerols

**Affiliations:** ^1^ Acute Medicine Department Queen Elizabeth the Queen Mother Hospital Margate UK

**Keywords:** drug‐induced lung disease, eosinophilic pneumonitis, mesalazine, non‐resolving pneumonia, ulcerative colitis

## Abstract

Mesalazine is widely used in the management of ulcerative colitis and is generally well tolerated. Rarely, it can cause eosinophilic pneumonitis, a potentially serious form of drug‐induced lung injury that often mimics infection. We report a 24‐year‐old male with longstanding ulcerative colitis on mesalazine therapy for 2 years and 4 months who presented with progressive dyspnea and non‐resolving pulmonary infiltrates. Despite empirical antibiotic therapy, his respiratory symptoms worsened. Investigations revealed marked peripheral eosinophilia and bilateral subpleural ground‐glass opacities on CT imaging. Mesalazine‐induced eosinophilic pneumonitis was suspected. Drug withdrawal and systemic corticosteroid therapy resulted in rapid clinical and radiological improvement. This case highlights that mesalazine‐related pulmonary toxicity may occur even after prolonged stable therapy and should be considered in patients with ulcerative colitis presenting with unexplained or non‐resolving respiratory symptoms.

## Key Clinical Message

Mesalazine can cause eosinophilic pneumonitis even after years of well‐tolerated therapy. In patients with ulcerative colitis presenting with non‐resolving respiratory symptoms, persistent eosinophilia and peripheral ground‐glass opacities should prompt consideration of drug‐induced lung injury. Early drug withdrawal enables rapid recovery.

## 1 Introduction

Mesalazine (5‐aminosalicylic acid) remains a cornerstone therapy in the management of ulcerative colitis [1]. Although generally considered safe, rare pulmonary adverse effects have been described, including interstitial pneumonitis, organizing pneumonia, and eosinophilic pneumonitis [2, 3, 4, 5].

These reactions are frequently under‐recognized because their clinical and radiological features overlap significantly with infectious and inflammatory lung diseases [6, 7].

Importantly, while most reported cases occur shortly after treatment initiation, delayed presentations after prolonged periods of apparent drug tolerance have been documented and are often overlooked. This diagnostic bias may result in inappropriate antimicrobial therapy and delayed recognition of drug‐induced lung injury [8].

## 2 Case History/Examination

A 24‐year‐old male with left‐sided ulcerative colitis, confirmed by prior endoscopic and histological assessment, presented with a 1‐week history of progressive exertional dyspnea and dry cough. He had been maintained on oral mesalazine (Pentasa) 2 g twice daily and mesalazine suppositories for 2 years and 4 months, with sustained clinical remission and no recent escalation of therapy.

The patient denied fever, hemoptysis, wheeze, recent travel, or infectious contacts. He was a lifelong non‐smoker and denied vaping, illicit drug use, or use of herbal or alternative supplements. There was no history of asthma, atopy, or occupational or environmental exposure.

On examination, he was tachycardic (120 beats per minute) with oxygen saturations of 95% on room air. Chest auscultation revealed reduced air entry at the right base and coarse inspiratory crackles on the left.

## 3 Differential Diagnosis, Investigations, and Treatment

### 3.1 Initial Blood Investigations Demonstrated

- White blood cell count: 20.6 × 10^9^/L
- C‐reactive protein: 205 mg/L
- Marked eosinophilia: 4.9 × 10^9^/L
- Normal renal and liver function (Table **1**).

**TABLE 1:** Summary of blood and microbiological investigations.

Investigations	Results	Reference range
White blood cell count (WBC)	20.6 × 10^9^/L	4.0–11.0 × 10^9^/L
Neutrophil count	10.7 × 10^9^/L	2.0–7.5 × 10^9^/L
C‐reactive protein (CRP)	205 mg/L	< 5 mg/L
Eosinophil count	4.9 × 10^9^/L	0.0–0.5 × 10^9^/L
Respiratory viral PCR	Negative	Negative
Urinary *Legionella* antigen	Negative	Negative
Urinary pneumococcal antigen	Negative	Negative
Antinuclear antibodies (ANA)	Negative	Negative
Anti‐neutrophil cytoplasmic antibodies (ANCA)	Negative	Negative
Blood cultures	No growth	No growth
Sputum culture	No growth	No growth

**FIGURE 1:**
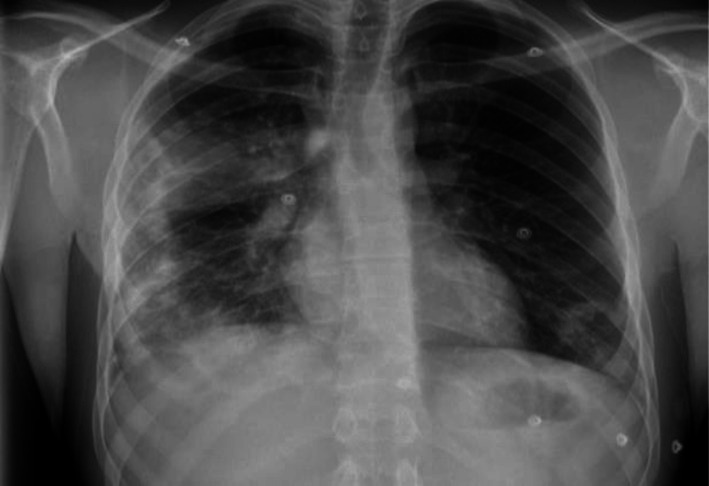
Initial chest radiograph showing bilateral patchy mid‐ and lower zone opacities, more prominent on the right. The diffuse, non‐lobar distribution is atypical for bacterial pneumonia and raised suspicion for a noninfective inflammatory process.

Chest radiography revealed bilateral patchy mid‐ and lower zone infiltrates (Figure 1). The patient was initially managed as community‐acquired pneumonia and discharged with oral antibiotics via a virtual ward pathway.

**FIGURE 2:**
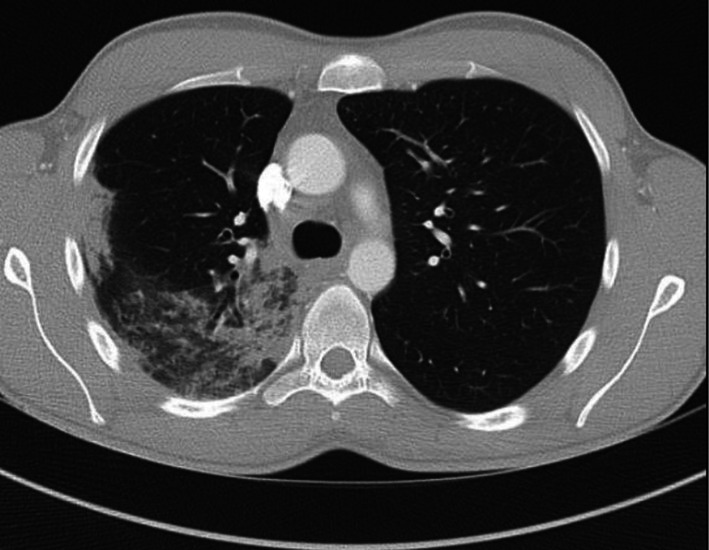
CT pulmonary angiogram demonstrates bilateral subpleural ground‐glass opacities with patchy consolidation. The peripheral distribution is characteristic of eosinophilic pneumonitis and hypersensitivity‐type lung injury rather than infection.

He re‐presented 3 days later with worsening dyspnea and pleuritic chest pain. CT pulmonary angiography excluded pulmonary embolism but demonstrated bilateral subpleural ground‐glass opacities with patchy consolidation (Figure 2).

An extensive microbiological work‐up, including respiratory viral PCR, blood cultures, and urinary pneumococcal and Legionella antigens, was negative. Repeat eosinophil count remained elevated at 4.6 × 10^9^/L.

Given the non‐resolving course, characteristic imaging pattern, and persistent eosinophilia, mesalazine‐induced eosinophilic pneumonitis was suspected. Mesalazine was discontinued, and oral prednisolone 30 mg daily was commenced following multidisciplinary discussion.

**FIGURE 3:**
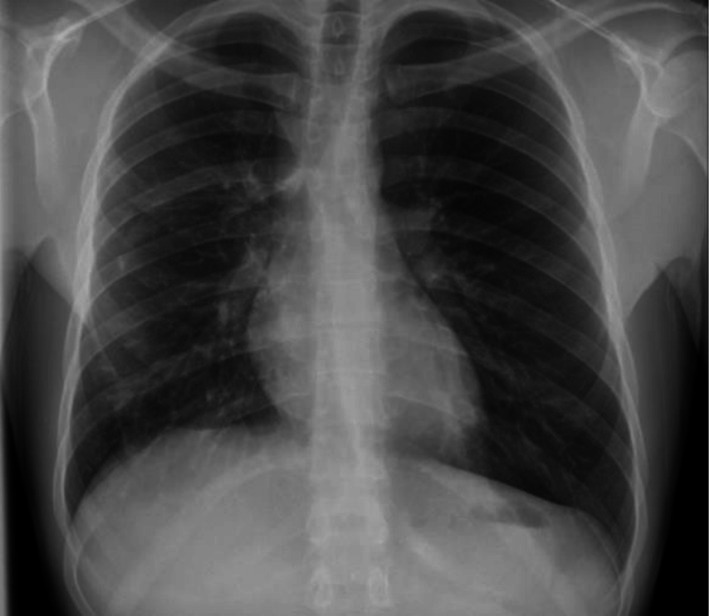
Follow‐up chest radiograph showing near‐complete resolution of bilateral infiltrates after cessation of mesalazine and initiation of systemic corticosteroid therapy.

The patient demonstrated marked clinical improvement within 72 h. Follow‐up imaging showed near‐complete radiological resolution (Figure 3). Pulmonary function testing performed 6 weeks later demonstrated normal spirometry and lung volumes, with no residual obstructive or restrictive defect.

Given the confirmed diagnosis of ulcerative colitis and concern for future relapse, he was transitioned to azathioprine for maintenance therapy, with no recurrence of respiratory symptoms.

## 4 Conclusion

Mesalazine‐induced eosinophilic pneumonitis should be considered in any patient with ulcerative colitis presenting with unexplained or non‐resolving respiratory symptoms, even after prolonged stable therapy. Recognition of eosinophilia and atypical imaging patterns is essential, as early drug withdrawal leads to rapid recovery and prevents irreversible lung injury [2, 3, 4, 6].

## 5 Discussion

Mesalazine‐induced eosinophilic pneumonitis is an uncommon yet clinically significant manifestation of drug‐induced lung disease in patients with inflammatory bowel disease [2, 3, 4]. Although mesalazine is widely regarded as a safe medication, fewer than 50 confirmed cases of eosinophilic pneumonitis have been reported in the literature [2, 3, 4]. This low number likely reflects under‐recognition rather than true rarity, as clinical presentation frequently mimics infectious pneumonia.

A major diagnostic challenge is the highly variable latency period between drug initiation and symptom onset [2, 3, 4]. While most reported cases occur within weeks or months of starting therapy, delayed presentations after several years of continuous treatment—as seen in this case—are documented but often overlooked. This case is therefore clinically important because it directly challenges a common diagnostic bias: the assumption that long‐term tolerance excludes drug toxicity.

The pathophysiological mechanism is thought to involve an idiosyncratic immune‐mediated hypersensitivity reaction, with T‐cell activation and eosinophilic infiltration of pulmonary tissue [5, 6].

Characteristic imaging features include bilateral peripheral or subpleural ground‐glass opacities, often accompanied by consolidation. Persistent peripheral eosinophilia is a critical diagnostic clue and should prompt evaluation for eosinophilic lung disease once infection is excluded [9]. These findings overlap with atypical infections, viral pneumonitis, organizing pneumonia, and eosinophilic granulomatosis with polyangiitis (EGPA) [6, 9].

Peripheral eosinophilia is a critical diagnostic marker and should prompt consideration of eosinophilic lung disease once common infectious causes have been excluded [2, 4]. The significant reduction in eosinophil count following mesalazine discontinuation further strengthened the causal association.

Although bronchoalveolar lavage may be useful in confirming eosinophilic alveolitis, it is not mandatory when the clinical, laboratory, radiological, and therapeutic responses are strongly supportive, as demonstrated in this case.

Important differentials include atypical infection, organizing pneumonia, eosinophilic granulomatosis with polyangiitis, and pulmonary manifestations of inflammatory bowel disease. The absence of asthma, vasculitic features, ANCA positivity, and the rapid response to drug withdrawal argue strongly against these alternatives in this case [8].

The cornerstone of management is immediate discontinuation of mesalazine. Corticosteroids remain the mainstay of treatment in moderate to severe cases due to their rapid anti‐inflammatory effects [5, 6]. Rechallenge with mesalazine or other 5‐ASA formulations is not recommended, given the risk of recurrence or more severe reactions [2, 4].

In addition to pulmonary toxicity, long‐term 5‐ASA therapy is associated with renal complications, including interstitial nephritis, necessitating regular blood and urine monitoring. Routine surveillance chest imaging is not recommended in asymptomatic patients; however, clinicians should maintain a low threshold for respiratory evaluation when new symptoms develop.

This case reinforces several important clinical lessons:

(i) delayed mesalazine‐induced pulmonary toxicity can occur even after years of stable therapy;

(ii) eosinophilia is a key diagnostic clue in non‐resolving pneumonia;

(iii) early drug withdrawal prevents progression to irreversible lung damage;

(iv) multidisciplinary collaboration is essential to avoid delayed diagnosis and inappropriate management.

## Author Contributions

**Siraj Nasim:** formal analysis, investigation, methodology, writing – original draft. **Tamer Mohamed Zaalouk:** data curation, project administration, supervision, visualization. **Abu Bocus:** formal analysis, software, validation. **Maged Kerols:** data curation, investigation, visualization.

## Funding

The authors received no financial support for the research, authorship, and/or publication of this article. The research was performed as part of the employment of the authors in QEQM hospital, Margate (EKUHFT).

## Consent

Written informed consent was obtained from the patient for publication of this case report.

## Conflicts of Interest

The authors declare no conflicts of interest.

## Data Availability

This case report does not include any publicly available datasets. All clinical data referenced were obtained from routine patient care and have been fully anonymized to protect patient confidentiality.
